# Supplementary material for: The Systems Biology Research Tool: evolvable open-source software
Source: BMC Syst Biol. 2008 Jun 29;2:55. doi: 10.1186/1752-0509-2-55 (PMC2446383; doi:10.1186/1752-0509-2-55)
Supplement: Additional file 1 — SBRT Archive. An archive of the current version of the Systems Biology Research Tool. [file 1752-0509-2-55-S1.zip › sbrt-1.4.0/doc/developers_guide/api/sbrt/kernel/math/algebra/class-use/LinearSystemSolution.html]

Uses of Interface sbrt.kernel.math.algebra.LinearSystemSolution


|  |  |  |
| --- | --- | --- |
| **Overview** \| **Package** \| **Class** \| **Use** \| **Tree** \| **Deprecated** \| **Index** \| **Help** | |  |
| PREV   NEXT | **FRAMES**    **NO FRAMES**     **All Classes** |  |


---


## **Uses of Interface sbrt.kernel.math.algebra.LinearSystemSolution**

| Packages that use LinearSystemSolution | |
| --- | --- |
| **sbrt.ext.mma.algebra** | Provides classes that allow communication with Mathematica for the purposes of algebra. |
| **sbrt.kernel.fba.comp** | Provides classes and interfaces for performing Flux Balance Analysis computations. |
| **sbrt.kernel.fba.proc** | Provides classes and interfaces for executing Flux Balance Analysis processes. |
| **sbrt.kernel.math.algebra** | Provides classes and interfaces for creating objects relevant to algebra. |
| **sbrt.kernel.math.algebra.proc** | Provides classes and interfaces for executing algebra-related processes. |
| **sbrt.kernel.math.geom** | Provides classes and interfaces for creating objects relevant to computational geometry. |
| **sbrt.shell.mng.fba** | Provides classes and interfaces for creating Flux Balance Analysis process managers. |

Uses of LinearSystemSolution in sbrt.ext.mma.algebra

| Methods in sbrt.ext.mma.algebra that return LinearSystemSolution | |
| --- | --- |
| `LinearSystemSolution<V>` | `MathematicaSolver.solve(java.util.Map<LinearComb<V>,java.lang.Double> equations)`             Computes and returns a solution to the provided system of linear equations. |

Uses of LinearSystemSolution in sbrt.kernel.fba.comp

| Methods in sbrt.kernel.fba.comp that return LinearSystemSolution | |
| --- | --- |
| `static LinearSystemSolution<java.lang.String>` | `FbaSolver.solve(Fluxome fluxome, LinearSystemSolver<java.lang.String> solver)`             Solves *Sv = 0*, where *S* is the stoichiometry matrix of the provided fluxome and *v* is the vector of fluxes.** |

Constructors in sbrt.kernel.fba.comp with parameters of type LinearSystemSolution

`CdHarFluxVectorComputer(IrrevRxnFluxome fluxome,
LinearSystemSolution<java.lang.String> systemSolution)`
  
          Constructs a new random flux vector computer for the provided fluxome.

`FastMsFluxRangeComputer(FbaOptComputer computer,
java.util.Map<java.lang.String,java.util.Set<LinearComb<java.lang.String>>> fluxCaps,
LinearSystemSolution<java.lang.String> systemSolution,
double zeroCutoff)`
  
          Constructs a new flux interval computer.

Uses of LinearSystemSolution in sbrt.kernel.fba.proc

| Method parameters in sbrt.kernel.fba.proc with type arguments of type LinearSystemSolution | |
| --- | --- |
| `void` | `FbaSolverProcess.run(ResultsManager<LinearSystemSolution<java.lang.String>> resultsManager)`             Solves Sv = 0 and adds the result to the provided manager. |

Uses of LinearSystemSolution in sbrt.kernel.math.algebra

| Classes in sbrt.kernel.math.algebra that implement LinearSystemSolution | |
| --- | --- |
| `class` | `HashLinearSystemSolution<V>`             This class is a concrete implemenation of `LinearSystemSolution`. |

| Methods in sbrt.kernel.math.algebra that return LinearSystemSolution | |
| --- | --- |
| `static <V> LinearSystemSolution<V>` | `HashLinearSystemSolution.simplify(LinearSystemSolution<V> solution, java.util.Map<? extends V,Interval> variableBounds, double tolerance)`             Returns a simplified version of the provided linear system solution given the variable bounds. |
| `LinearSystemSolution<V>` | `HashLinearSystemSolution.simplify(java.util.Map<?,java.lang.Double> variableValues, double tolerance)`             Computes and returns a simplified solution by plugging in the provided values. |
| `LinearSystemSolution<V>` | `LinearSystemSolution.simplify(java.util.Map<?,java.lang.Double> variableValues, double tolerance)`             Computes and returns a simplified solution by plugging in the provided values. |
| `LinearSystemSolution<V>` | `LinearSystemSolver.solve(java.util.Map<LinearComb<V>,java.lang.Double> equations)`             Computes and returns a solution to the provided system of linear equations. |

Methods in sbrt.kernel.math.algebra with parameters of type LinearSystemSolution

`static

<V> LinearSystemSolution<V>`

`HashLinearSystemSolution.simplify(LinearSystemSolution<V> solution,
java.util.Map<? extends V,Interval> variableBounds,
double tolerance)`
  
          Returns a simplified version of the provided linear system solution
given the variable bounds.

Uses of LinearSystemSolution in sbrt.kernel.math.algebra.proc

| Method parameters in sbrt.kernel.math.algebra.proc with type arguments of type LinearSystemSolution | |
| --- | --- |
| `void` | `LinearSolverProcess.run(ResultsManager<LinearSystemSolution<V>> resultsManager)`             Solves the system of linear equations and adds the solution to the provided results manager. |

Uses of LinearSystemSolution in sbrt.kernel.math.geom

| Methods in sbrt.kernel.math.geom that return LinearSystemSolution | |
| --- | --- |
| `LinearSystemSolution<V>` | `HashConvexPolytopeOld.getSystemSolution()`             Returns the linear system solution used to form this convex polytope. |

Methods in sbrt.kernel.math.geom with parameters of type LinearSystemSolution

`static

<V> HashConvexPolytope<V>`

`HashConvexPolytope.make(LinearSystemSolution<V> systemSolution,
java.util.Map<? extends V,Interval> variableBounds,
double tolerance)`
  
          Constructs a new convex poltyope from the provided linear system solution
and variable bounds.

Constructors in sbrt.kernel.math.geom with parameters of type LinearSystemSolution

`HashConvexPolytopeOld(LinearSystemSolution<V> systemSolution,
java.util.Map<? extends V,Interval> variableBounds,
double tolerance)`
  
          Constructs a new convex poltyope from the provided linear system
solution and variable bounds.

Uses of LinearSystemSolution in sbrt.shell.mng.fba

| Methods in sbrt.shell.mng.fba that return LinearSystemSolution | |
| --- | --- |
| `static LinearSystemSolution<java.lang.String>` | `FbaManager.getFluxomeSolution(ManagerInput<java.lang.String,java.lang.String> input, java.io.PrintWriter out, Fluxome fluxome)`             Opens the file denoted by `FbaManager.SYSTEM_SOLUTION_FILE` in the provided manager input and returns the linear system solution it contains. |

---


|  |  |  |
| --- | --- | --- |
| **Overview** \| **Package** \| **Class** \| **Use** \| **Tree** \| **Deprecated** \| **Index** \| **Help** | |  |
| PREV   NEXT | **FRAMES**    **NO FRAMES**     **All Classes** |  |


---
